# Detecting false intent using eye blink measures

**DOI:** 10.3389/fpsyg.2013.00736

**Published:** 2013-10-11

**Authors:** Frank M. Marchak

**Affiliations:** Veridical Research and Design CorporationBozeman, MT, USA

**Keywords:** credibility assessment, false intent, blink, oculometrics, deception detection

## Abstract

Eye blink measures have been shown to be diagnostic in detecting deception regarding past acts. Here we examined—across two experiments with increasing degrees of ecological validity—whether changes in eye blinking can be used to determine false intent regarding future actions. In both experiments, half of the participants engaged in a mock crime and then transported an explosive device with the intent of delivering it to a “contact” that would use it to cause a disturbance. Eye blinking was measured for all participants when presented with three types of questions: relevant to intent to transport an explosive device, relevant to intent to engage in an unrelated illegal act, and neutral questions. Experiment 1 involved standing participants watching a video interviewer with audio presented ambiently. Experiment 2 involved standing participants questioned by a live interviewer. Across both experiments, changes in blink count during and immediately following individual questions, total number of blinks, and maximum blink time length differentiated those with false intent from truthful intent participants. In response to questions relevant to intent to deliver an explosive device vs. questions relevant to intent to deliver illegal drugs, those with false intent showed a suppression of blinking during the questions when compared to the 10 s period after the end of the questions, a lower number of blinks, and shorter maximum blink duration. The results are discussed in relation to detecting deception about past activities as well as to the similarities and differences to detecting false intent as described by prospective memory and arousal.

## Introduction

Interest in determining veracity has a long history, with documented methodologies extending back to at least 900 BC [for a review, see Trovillo (1939a,b)]. The majority of these efforts have focused on the detection of deception, a term which has a variety of characterizations (Masip et al., 2004). A widely accepted definition of deception is provided by Vrij (2008) as “a successful or unsuccessful deliberate attempt, without forewarning, to create in another a belief which the communicator considers to be untrue” (p. 15). While the temporal period during which this untruth is committed is unspecified, the majority of research has focused on detecting indicators of concealed past behavior.

The question arises concerning the ability of determining whether an individual is being deceptive regarding future intentions. In contrast to knowledge of past activities, intent involves a goal or plan of action for the future, in which both the execution of the action and its outcome are uncertain. As such, intent may be defined as “a person's mental representations of his/her planned future actions” (Vrij et al., 2011a), and by extension, false intent involves misleading others regarding upcoming but not yet realized actions.

There have been efforts recently to determine if an individual is misleading others regarding the true purpose, or intent, of their future actions. Vrij and colleagues (2011a); Vrij and his colleagues (2011b) have shown differences in verbal responses to questions between truthful intent participants and those with false intent in terms of number of words, level of details, plausibility, contradictions, and corrections. Meixner and Rosenfeld (2011), using a P300-based concealed information test, were able to detect with a high level of accuracy individuals who planned a mock terrorism attack. Aikins et al. (2010) examined changes in respiratory sinus arrhythmia (RSA) in participants that were either truthful or were to respond deceptively about a future mock crime. The data showed greater reductions in RSA during testing of participants that were deceptive regarding an upcoming task compared to the truthful intent participants.

Since being untruthful regarding both past and future acts includes the attribute of a desire to mislead, it has been hypothesized that cues indicative of false intent arise from analogous emotional, cognitive, and behavioral processes involved in deception (Martin et al., 2007). Traditional deception detection techniques focus on changes in measures of autonomic functions—such as respiration, cardiac activity, and electrodermal activity—as indicators of deceptive responses regarding previous activities (Abrams, 1989). However, other measures, such as changes in ocular parameters including eye movements, pupil diameter, and blink rate, have been examined as possible alternative markers of deception (e.g., Marchak et al., 2011; Cook et al., 2012).

One of the first examinations of these indices was conducted by Cutrow et al. (1972). Evaluating multiple physiological measures, including eye blinks, they concluded that eye blink rate decreases under circumstances of lying. Fukuda (2001) investigated the temporal distribution of eye blinks while subjects performed a guilty knowledge test (GKT) with playing cards. It was found that more eye blinks occurred before responses following presentation of the relevant card while more eye blinks occurred after responses for presentation of irrelevant cards. DePaulo et al. (2003) reviewed the literature on deception detection and identified over 100 cues to deception. Of relevance here, they found that blinking was more prevalent when lies involved transgressions, discovery of which could have serious consequences. In another study employing emotionally arousing stimuli in a GKT, Thonney et al. (2005) found a difference in GKT eye blinking scores for emotional stimuli over neutral stimuli.

Leal and Vrij (2008) examined blink rates in liars and truth tellers during and after verbal recall of events and found that liars showed a decrease in blink rate during deception as compared with a baseline period and an increase in blink rate in the period following the telling of the lie. In a study of the GKT with the same subjects (Leal and Vrij, 2010) they found that liars exhibited a lower blink rate in response to key items as compared to control items, but there was no difference for truth tellers.

Taken together, these findings suggest that blink parameters are diagnostic in determination of deception regarding past actions. These differences can be explained both by theories of cognitive load (e.g., Fogarty and Stern, 1989; Fukuda et al., 2005; Irwin and Thomas, 2010) as well as arousal-based theories (e.g., Stern, 1992). While it is difficult to isolate the specific cause of the differences in blink behavior between liars and truth tellers, it appears that the findings are reliable and repeatable.

The question arises if these same measures can be used to determine whether an individual is being deceptive regarding future intentions as opposed to past actions. The present work attempted to determine if changes in eye blink parameters could be used to detect false intent. Based on the findings of differences in blink parameters between truthful individuals and those deceptive about prior actions, the primary hypothesis is that individuals with false intent will exhibit a suppression of blink rate during intent relevant questions, accompanied by a rebound in blink rate in the period following the question end, as well as a lower overall number of blinks and shorter blink durations when compared to those with truthful intentions. This effect was examined in two experiments that manipulated ecological validity between a controlled, standardized prerecorded video presentation of questions and questions presented by a live interviewer.

## Experiment 1

### Methods

#### Participants

Participants (*N* = 54) were recruited through advertisements in a local newspaper and through an online classified advertising site. A total of 25 (9 female/16 male; average age = 27.76, *SD* = 8.83) participated in the false intent condition and 29 (12 female/17 male; average age = 28.05, *SD* = 9.05) in the truthful intent condition. The experimental design and data collection procedures were reviewed and approved by the Montana State University Human Subjects Committee and informed consent was obtained from all subjects.

#### Apparatus

Pupil diameter, blink, and eye movement data were collected using a Smart Eye Pro version 5.4 remote eye tracker. The system has a 60 Hz sampling frequency and is capable of achieving pupil measurement accuracy to.01 mm. Voice responses were collected using a Cedrus SV-1 Voice Key.

Pre-recorded auditory instructions and questioning information were presented as sound files using a TDT System 3 Psychoacoustic Workstation through Altec Lansing VS2120 amplified speakers. Video was presented on a LaCie 324 LCD monitor, a 24-inch widescreen display located 60 cm from the participant. Data from all sources were time-stamped and synchronized through a Cambridge Research Systems ViSaGe, a multiple I/O interface, timing and visual stimulus generation device.

#### Procedure

Participants responding to the recruitment advertisements were directed to call a local phone number and were presented with a voicemail message instructing them to leave a name and number where they could be contacted.

Four offices in three local buildings were used. Participants first reported to an intake office in a facility that houses professional businesses where they completed an Institutional Review Board (IRB) consent form and were provided directions to the location of another building, within walking distance, where the participant was to receive further instructions.

The test site was located in a building approximately ½block away from the intake office. Participants walked independently to the building and entered the instruction room. Each participant was randomly assigned to a false intent or truthful intent condition. In both conditions, participants listened to taped instructions through headphones and were told that in addition to the $25 USD payment for participation they could earn an addition $25 USD for successfully passing a credibility assessment test. Participants in the false intent condition heard instructions in which they were to commit a mock crime by taking a “fuse lighter” from a downstairs office in the building and providing it to a “contact” after completing a credibility assessment test at another location. Documentation taken from Pickett (1999) was provided illustrating the fuse-lighter as was a photograph of the fictitious “contact.” In the truthful intent condition, participants heard instructions in which they were to remove a note from the door of a downstairs office and were not provided with the supplementary materials.

In both conditions, participants exited the instruction room, walked around the block, and entered the building through a side door. They then proceeded downstairs to a basement office. Those in the truthful intent condition simply removed a sticker containing numbers from the door. Those in the false intent condition were required to enter the office and find and remove the fuse lighter. The office containing the fuse-lighter was furnished to resemble a working facility.

In both conditions, the participants exited the building through a third door and proceeded approximately 2 blocks to our laboratory to take a credibility assessment examination. Details of the examination and data collection are provided in the next section.

### Data collection

After arriving at the laboratory, both false intent and truthful intent participants were questioned about the event using a Relevant-Relevant comparison test format (RRT). Based on a paradigm developed by Kircher and Raskin (2006), the RRT consists of two sets of relevant questions as well as neutral questions (N). One set of relevant questions addressed matters related to plans to transport illegal drugs for delivery to another person after the credibility assessment test (Drug Intent) while the other set of relevant questions was related to plans to deliver a fuse lighter to a contact after the credibility assessment test (Explosives Intent). Both sets of relevant planned actions were to occur after the questioning and the questions asked about future actions, but only the Explosive Intent was carried out by those in the false intent condition. Table 1 provides a list of the questions employed and question durations in milliseconds.

**Table 1:** **Test questions and question durations**.

Do you live in Bozeman or a surrounding community? (Neutral)	3005 ms
Do you intend to transport illegal drugs today? (Drug Intent)	2610 ms
Were you born between nineteen twenty and nineteen ninety? (Neutral)	3020 ms
Do you plan to help cause a disturbance today? (Explosives Intent)	2420 ms
Did you attend elementary school in the United States? (Neutral)	2890 ms
Are you attempting to transport part of an explosive device? (Explosives Intent)	3520 ms
Is today's date Sunday, February eighth two thousand and nine? (Neutral)	3610 ms
Are you attempting to smuggle illegal drugs today? (Drug Intent)	2670 ms
Are you in Bozeman, Montana at this moment? (Neutral)	2280 ms
Do you plan to provide a fuse lighter to someone today? (Explosives Intent)	3060 ms
Did you use public transportation to arrive here today? (Neutral)	2960 ms
Do you plan to provide illegal drugs to another person? (Drug Intent)	2910 ms

In contrast to the Comparison Question Test (CQT) format (Abrams, 1989), in which responses to relevant questions are compared to responses regarding general undesirable acts (e.g., “Before the age of 18, did you ever take something that didn't belong to you?”), the RRT compares responses to two types of relevant questions—one directed toward the area of interest and the other toward an equally relevant area but one in which the examinee has not engaged. In both test formats, neutral questions are interspersed between the questions of interest to serve as buffers and permit the examinee's physiological reactions to return to baseline levels. In the RRT, those in the truthful intent condition should have similar responses to both of the relevant question types, while those in the false intent condition should have different reactions to the explosives intent questions compared to the drug intent questions.

In both truthful intent and false intent conditions, participants stood facing a video monitor and questions were presented aurally through recordings over speakers simultaneously with a prerecorded video of an interviewer asking the questions. Ocular-based parameters were measured in an ambient illumination-controlled room (15.1 lux above). Participants responded “yes” or “no” verbally into a microphone and response time was recorded, but not used in the current analyses due to poor reliability of the data collection device. Question start and question end was marked by a time stamp in the video synchronized with the ocular data. Each question was followed by a 15 s interval before the next question was presented.

After completing the data collection process, participants were debriefed. Those in the false intent condition were told that they did not need to meet a contact, the experiment was completed, and they were asked to return the fuse lighter. Participants in both conditions were told that they would receive the $25 USD bonus. They were then paid and thanked for their participation.

### Data processing

For each participant, ocular data were time-stamped and synchronized with the video and audio presentation of the questions. Blinks were identified from the eye tracker data as intervals of 60–1000 ms where the pupil diameter was equal to zero. Three measures were calculated from these data. Blink Count Difference was determined as the number of blinks in the period from the end of a question to 10 s after question end minus the number of blinks during the question presentation. This serves as a measure of blink suppression during question presentation. The use of the 10 s time period was suggested by Stern (pers. Commun., November 20, 2008) based on his experience and was verified through pilot testing that examined time intervals from 5 to 20 seconds (*N* = 8). The average duration of all questions was 2912.92 ms, while the average durations for the Drug Intent, Explosives Intent, and Neutral questions were 2730 ms, 3000 ms, and 2960.8 ms, respectively. Number of blinks was the total number of blinks during the question and the 10 s period following the question end. Maximum blink duration was the length in milliseconds of the longest blink time during the analysis period for each question.

### Results

All participants verbally responded “yes” or “no” to all questions and none of the responses were eliminated from analysis. Table 2 presents the raw means and standard deviations of the blink count difference, number of blinks, and maximum blink duration for Drug Intent, Explosives Intent, and Neutral questions for participants in both the false intent and truthful intent conditions. The ocular-based data were normalized by calculating *z*-scores and submitted to a repeated measures multivariate analysis of variance (RMANOVA). For Drug Intent vs. Explosives Intent questions, there were significant within-subject multivariate effects for Relevance × Intent condition, *F*_(3, 50)_ = 3.908, *p* = 0.014, η^2^_*p*_ = 0.190. There were significant within-subjects effects of Blink Count Difference, *F*_(1, 52)_ = 6.213, *p* = 0.016, η^2^_*p*_ = 0.107, and Number of Blinks *F*_(1, 52)_ = 7.096, *p* = 0.010, η^2^_*p*_ = 0.120. Maximum Blink Duration approached significance _(1, 52)_ = 3.526, *p* = 0.066, η^2^_*p*_ = 0.064.

**Table 2:** **Raw means and standard deviations for blink count difference, number of blinks, and maximum blink duration (ms) by question type—Experiment 1**.

**Measure**	**Drug intent**		**Explosives intent**		**Neutral**	
	**Mean**	***SD***	**Mean**	***SD***	**Mean**	***SD***
**BLINK COUNT DIFFERENCE**						
False intent	0.183	0.182	0.134	0.193	0.244	0.172
Truthful intent	0.232	0.205	0.279	0.224	0.266	0.204
**NUMBER OF BLINKS**						
False intent	5.107	3.661	4.467	2.693	5.882	3.319
Truthful intent	5.805	3.999	6.368	4.438	6.823	4.303
**MAXIMUM BLINK DURATION**						
False intent	210.000	149.505	177.760	109.814	221.700	136.474
Truthful intent	201.741	122.883	212.126	119.379	231.398	123.879

**Figure 1:**
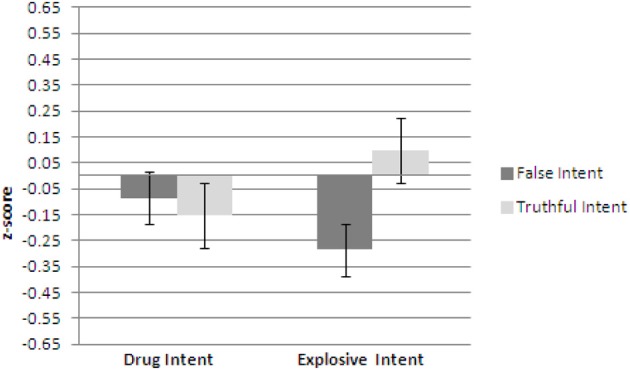
**Experiment 1—Blink count difference as the number of blinks in the period from the end of a question to 10 s after question end minus the number of blinks during the question presentation for false intent and truthful Intent participants.** Error bars show Standard Error.

**Figure 2:**
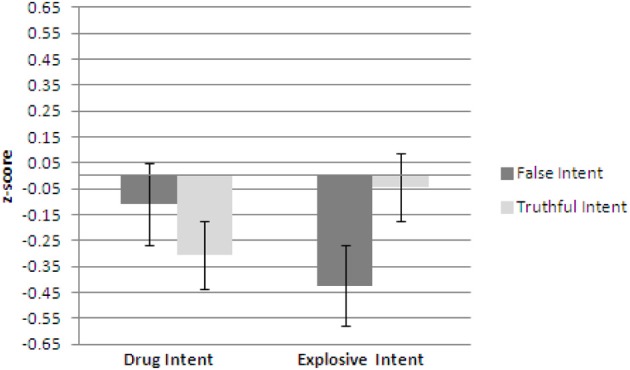
**Experiment 1—Number of blinks for false intent and truthful intent participants.** Error bars show Standard Error.

**Figure 3:**
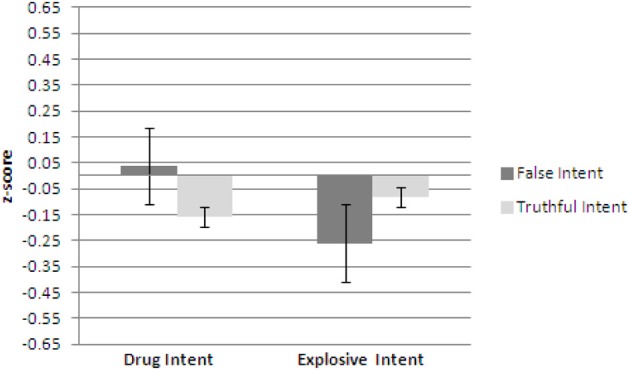
**Experiment 1—Maximum blink duration for false intent and truthful intent participants.** Error bars show Standard Error.

Figures 1–3 show plots of normalized Blink Count Difference, Number of Blinks, and Maximum Blink Duration, respectively, for Drug Intent and Explosives Intent questions for participants in both the false intent and truthful intent conditions. Participants in the false intent condition showed a lower blink count difference, fewer numbers of blinks, and shorter but not significantly different maximum blink duration for the Explosives Intent vs. the Drug Intent questions.

In order to examine the relationship between the neutral and relevant questions, the normalized data for the Neutral, Drug Intent, and Explosives Intent questions were submitted to a RMANOVA. There were significant within-subject multivariate effects for Relevance × Intent condition, *F*_(6, 47)_ = 2.585, *p* = 0.030, η^2^_*p*_ = 0.248. There were significant within-subjects effects of Blink Count Difference, *F*_(2, 104)_ = 5.328, *p* = 0.006, η^2^_*p*_ = 0.093, and Number of Blinks *F*_(2, 104)_ = 4.540, *p* = 0.014, η^2^_*p*_ = 0.080. Maximum Blink Duration was not significant *F*_(2, 104)_ = 1.846, *p* = 0.163.

To examine the effect of age and gender, the data were analyzed in the same manner as above but with age and gender as between subject variables. For Drug Intent vs. Explosives Intent questions, there were no significant effects for Gender by Age, *F*_(21, 45)_ = 1.140, *p* = 0.346, Age by Intent, *F*_(12, 45)_ = 1.678, *p* = 0.104, or Gender by Intent, *F*_(3, 13)_ = 1.727, *p* = 0.211.

Tables 3, 4 show the results of a discriminant analysis using all data and a leave-one-out procedure, respectively, to investigate how well the three blink parameters successfully classify false intent and truthful intent individuals. Using all the data, 72.4% of the truthful intent participants and 64.0% of those with false intent were correctly classified, with an overall correct classification rate of 68.5%. Results of the leave-one-out analysis found 72.4% of the truthful intent participants and 60.0% of those with false intent correctly classified, with an overall correct classification rate of 67.6%.

**Table 3:** **Discriminant analysis classification results—Experiment 1**.

**Actual**	**Predicted**		**Overall (%)**
	**Truthful intent (%)**	**False intent (%)**	
Truthful intent	72.4	27.6	
False intent	36.0	64.0	68.5

**Table 4:** **Discriminant analysis leave-one-out classification results—Experiment 1**.

**Actual**	**Predicted**		**Overall (%)**
	**Truthful intent (%)**	**False intent (%)**	
Truthful intent	72.4	27.6	
False intent	40.0	60.0	67.6

### Discussion

Participants with false intent showed a significantly lower blink count difference and lower number of blinks for the Explosive Intent questions as compared to the Drug Intent questions, with the difference in maximum blink duration approaching significance, as compared to participants with truthful intent. There were no significant differences due to age or gender.

## Experiment 2

### Methods

#### Participants

Participants (*N* = 57) were recruited through the same avenues used in Experiment 1. A total of 29 (11 female/18 male; average age = 26.06, *SD* = 9.77) participated in the false intent condition and 28 (9 female/19 male; average age = 33.40, *SD* = 13.18) participated in the truthful intent condition. The experimental design and data collection procedures were reviewed and approved by the Montana State University Human Subjects Committee and informed consent was obtained from all subjects.

#### Apparatus

The same equipment and set up used in Experiment 1 was employed with the following changes. The video monitor was replaced with a podium located in front of the participant and a live interviewer located 60 cm from the participant. A Mimo IMO touch screen input display was used to present the question text to the interviewer. A push button was used by the interviewer to time stamp the question start and question end and synchronize with the ocular data. The voice key was not used.

#### Procedure

The procedure and instructions were the same as in Experiment 1.

### Data collection

The data collection process was similar to that in Experiment 1. After arriving at the laboratory, participants stood before the podium and a research assistant performed a short calibration procedure for the eye tracker that involved having the participant fixate on five spots located in front of them. When calibration was complete, the interviewer entered and sat behind the podium. Questions were presented to the interviewer on a Mimo IMO touch screen display, which also indicated the inter-question interval time before the displaying the next question. Each question was read aloud. When the interviewer was ready, he pushed a hand-held button to code question start time into the data stream. After reading the question, question end time was also marked by the interviewer pressing a button. When the 15 s inter-trial interval had passed, the display presented the next question to the interviewer.

### Data processing

Data processing was identical to Experiment 1.

### Results

All participants verbally responded “yes” or “no” to all questions and none of the responses were eliminated from analysis. Table 5 presents the raw means and standard deviations of the blink count difference, number of blinks, and maximum blink duration for Drug Intent, Explosives Intent, and Neutral questions for participants in both the false intent and truthful intent conditions. The ocular-based data were normalized by calculating z-scores and submitted to a repeated measures multivariate analysis of variance (RMANOVA). For Drug Intent vs. Explosives Intent questions, there were significant within-subject multivariate effects for Relevance × Intent Condition, *F*_(3, 53)_ = 10.362, *p* = 0.000, η^2^_*p*_ = 0.370. There were significant within-subjects effects of Blink Count Difference, *F*_(1, 55)_ = 12.983, *p* = 0.001, η^2^_*p*_ = 0.191, Number of Blinks *F*_(1, 55)_ = 20.156, *p* = 0.000, η^2^_*p*_ = 0.268, and Maximum Blink Duration (1, 55) = 18.179, *p* = 0.000, η^2^_*p*_ = 0.248.

**Table 5:** **Raw means and standard deviations for blink count difference, number of blinks, and maximum blink duration (ms) by question type—Experiment 2**.

**Measure**	**Drug intent**		**Explosives intent**		**Neutral**	
	**Mean**	***SD***	**Mean**	***SD***	**Mean**	***SD***
**BLINK COUNT DIFFERENCE**						
False intent	0.251	0.276	0.302	0.258	0.288	0.269
Truthful intent	0.321	0.224	0.229	0.221	0.256	0.192
**NUMBER OF BLINKS**						
False intent	4.219	3.785	5.130	4.349	4.850	4.520
Truthful intent	4.633	3.201	3.933	3.460	4.634	3.661
**MAXIMUM BLINK DURATION**						
False intent	239.958	113.008	285.505	125.791	245.495	97.888
Truthful intent	217.567	87.608	194.067	71.835	234.250	79.868

**Figure 4:**
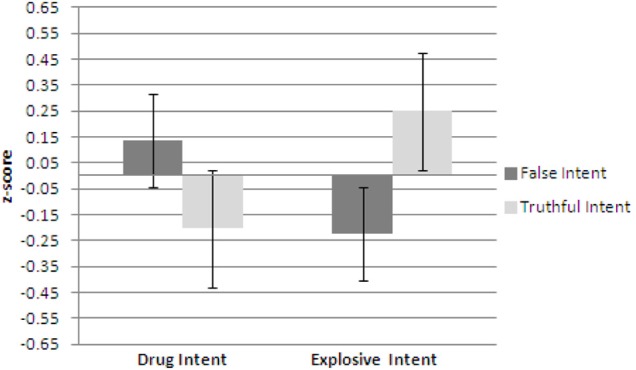
**Experiment 2—Blink count difference as the number of blinks in the period from the end of a question to 10 s after question end minus the number of blinks during the question presentation for false intent and truthful intent participants.** Error bars show Standard Error.

**Figure 5:**
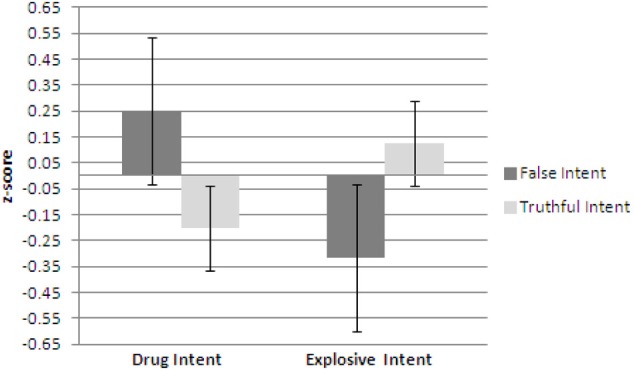
**Experiment 2—Number of blinks for false intent and truthful intent participants.** Error bars show Standard Error.

**Figure 6:**
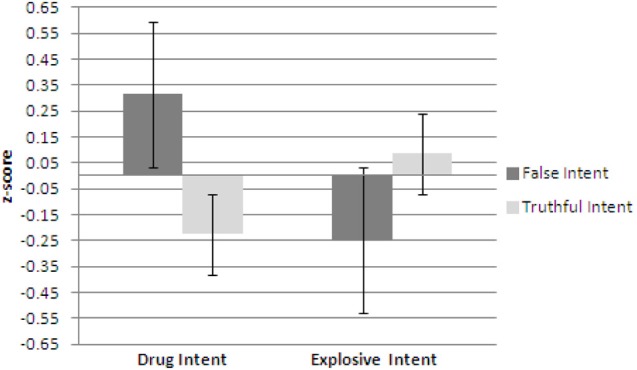
**Experiment 2—Maximum blink duration for false intent and truthful intent participants.** Error bars show Standard Error.

Figures 4–6 show plots of normalized Blink Count Difference, Number of Blinks, and Maximum Blink Duration, respectively, for Drug Intent and Explosives Intent questions for participants in both the false intent and truthful intent conditions. Participants in the false intent condition showed a lower blink count difference, fewer numbers of blinks, and shorter maximum blink duration for the Explosives Intent vs. the Drug Intent questions when compared to participants in the truthful intent condition.

In order to examine the relationship between the neutral and relevant questions, the normalized data for the Neutral, Drug Intent, and Explosives Intent questions were submitted to a RMANOVA. There were significant within-subject multivariate effects for Relevance × Intent condition, *F*_(6, 53)_ = 5.696, *p* = 0.000, η^2^_*p*_ = 0.406. There were significant within-subjects effects of Blink Count Difference, *F*_(2, 110)_ = 7.448, *p* = 0.001, η^2^_*p*_ = 0.119, and Number of Blinks *F*_(2, 110)_ = 9.186, *p* = 0.000, η^2^_*p*_ = 0.143. Maximum Blink Duration was significant *F*_(2, 110)_ = 10.13, *p* = 0.000, η^2^_*p*_ = 0.156.

To examine the effect of age and gender, the data were analyzed in the same manner as above but with age and gender as between subject variables. For Drug Intent vs. Explosives Intent questions, there were no significant effects for Gender by Age, *F*_(18, 51)_ = 0.763, *p* = 0.731, Age by Intent, *F*_(15, 51)_ = 1.012, *p* = 0.459, or Gender by Intent, *F*_(3, 15)_ = 0.131, *p* = 0.940.

Tables 6, 7 show the results of a discriminant analysis using all data and a leave-one-out procedure, respectively, to investigate how well the three blink parameters successfully classify false intent and truthful intent individuals. Using all the data, 72.0% of the truthful intent participants and 78.1% of those with false intent were correctly classified, with an overall correct classification rate of 75.4%. Results of the leave-one-out analysis found 68.0% of the truthful intent participants and 78.1% of those with false intent correctly classified, with an overall correct classification rate of 73.7%.

**Table 6:** **Discriminant analysis classification results—Experiment 2**.

**Actual**	**Predicted**		**Overall (%)**
	**Truthful intent (%)**	**False intent (%)**	
Truthful intent	72.0	28.0	
False intent	21.9	78.1	75.4

**Table 7:** **Discriminant analysis leave-one-out classification results—Experiment 2**.

**Actual**	**Predicted**		**Overall (%)**
	**Truthful intent (%)**	**False intent (%)**	
Truthful intent	68.0	32.0	
False intent	21.9	78.1	73.7

### Discussion

Similar to Experiment 1, participants with false intent showed a significantly lower blink count difference, lower number of blinks, and lower maximum blink duration for the Explosives Intent questions as compared to Drug Intent questions. However, there were differences in the responses to the Drug Intent questions as compared to those found in Experiment 1. One possible explanation for these differences could be related to the difference of being questioned by a videotaped interviewer and interacting with a live interviewer. While the questions and procedures were the same, participants in Experiment 1 did not have direct interactions with a live person. Riby et al. (2012) found a difference in skin conductance level (SCL) and increased arousal to live faces compared to video-mediated faces. This increase in arousal as a result of interacting with a live interviewer could contribute to the differences found in blink parameters between the two experiments.

## General discussion

The goal of this effort was to determine if variations in blink measures could differentiate between those with false intent and truthful intent individuals. Two experiments with differing degrees of ecological validity were conducted using either a prerecorded interviewer presented on a computer monitor or a live interviewer.

To date, the majority of research on determining false intent has employed verbal or non-verbal cues. Vrij and colleagues (2011a); Vrij and his colleagues (2011b) successfully detected false intent employing a structured interview and analysis of the resulting transcripts, as well as based on speech cues and participant willingness to be photographed. Similarly, Clemens et al. (2011) demonstrated that strategic interviewing elicits reliable cues to detecting false intent.

Only one study on detecting false intent has examined the use of physiological cues. Aikins et al. (2010) detected false intent by examining respiratory sinus arrhythmia (RSA)—an indicator of autonomic response—showing that individuals with false intent displayed decreased RSA compared to individuals with true intentions.

In both experiments reported here, it was found that for questions relevant to the harmful act to be committed, those with false intent showed a lower blink count difference, fewer numbers of blinks, and shorter maximum blink duration for questions related to their intent compared to questions related to another act for which they had no intent. These findings are consistent with previous findings in the literature that used blink measures to determine deception regarding past activities. While these analyses focused on factors related to blink counts and time, it would be possible to examine additional measures such as blink waveforms (Stern et al., 1984).

Two factors could contribute to the findings of differences in blink parameters of those with false intent: cognitive load and arousal. Both theories of cognitive load (e.g., Fogarty and Stern, 1989; Fukuda et al., 2005; Irwin and Thomas, 2010) as well as arousal-based theories (e.g., Stern, 1992) have been implicated in the context of deception detection.

As noted by Vrij et al. (2011a,b), differences between liars and truth tellers in both deception about past activities and future intentions are potentially affected by the increased cognitive load brought on in the untruthful situation. The effect of cognitive load on deception detection has been documented (e.g., Vrij et al., 2006; Leal et al., 2008). The effect of cognitive load on intention can be examined from two perspectives of memory about future events: episodic future thought (EFT) and prospective memory.

Granhag and Knieps (2011) have proposed that EFT is the central mental process involved in forming an intention and have used this framework to propose that the activation of a mental image in the pre-experiencing of an intention will be stronger for a true vs. a false intention. The current study did not explicitly test this construct so no comment can be made on its applicability based on the available data.

Prospective memory is defined as the process that permits remembering to engage in an intended action at some particular point in the future (Kvavilashvili and Ellis, 1996). Kliegel et al. (2000) describe prospective memory as consisting of three processes: developing a plan, remembering the plan, and remembering to execute the plan at some future time. In the current study, those with false intent had a plan to meet a contact and deliver the fuse lighter after taking the credibility assessment test, so were engaged in the first two processes but were stopped before the opportunity to begin the third process.

No studies were found that examined physiological measures of prospective memory with the exception of Hartwig et al. (2013), who examined gaze behavior to determine different approaches employed in solving prospective memory tasks. Hartwig et al. (2013) used the skewness of Voronoi cell distributions of fixation densities to quantify viewing strategies (Velichkovsky, 1999). Over et al. (2006) demonstrated that different visual tasks can be differentiated by skewness differences in the Voronoi cell sizes, and that tasks involving the same behavior would have similar skewness. Hartwig et al. (2013) found that when a prospective memory task was missed, participants exhibited gaze behavior similar to that seen in free viewing, including differential attention to details over only a few areas of interest. This viewing behavior resulted in a few large Voronoi cells and multiple small cells. If the prospective memory task was solved successfully, gaze behavior took on characteristics somewhat between those seen in free viewing and those seen in visual search, which was characterized by a large number of fixations across the entire display and many small Voronoi cells. These findings seem to imply that different approaches and levels of cognitive effort are involved in carrying out a prospective memory task and that the different processes are reflected in the ocular measures.

The effect of arousal on eye blink behavior has been investigated by Tanaka (1999) who examined the changes in blink rate, amplitude, and duration as a function of arousal level and found differences between a high arousal vigilance task and a low arousal counting task. Thonney et al. (2005) used experimentally aroused emotions of remorse and guilt and examined the effect of eye blinking and electrodermal response on Guilty Knowledge Test accuracy. They found that eye blinking was diagnostic for only the treatment group but not as accurate as electrodermal measures.

These findings have implications for further research on both blink measures and determination of false intent. One issue involves the contribution of cognitive workload and arousal to the changes in blink behavior. Both have been shown to affect blink rate, and while recent research has suggested that such findings are due primarily to cognitive load, neither this work nor previous efforts (Fukuda, 2001; Leal and Vrij, 2008, 2010) have explicitly addressed this question. Thus, no definitive conclusions may be drawn regarding the specific contributions of arousal and cognitive load to the findings.

Another factor of interest is distinguishing false intent about future actions (i.e., plan to deliver object to contact) from lying about past actions (i.e., the mock crime). In a standard polygraph examination employing, for example, the Comparison Question Technique, participants are asked a series of questions, a subset of which are relevant to the past act, such as the mock crime. Here, participants were asked questions relevant to their upcoming actions—delivering an explosive device to a contact that would use it to cause a disturbance—to be completed in the future after the questioning. None of the questions referred to activities previously performed.

Vrij et al. (2011b) explicitly compared differences in verbal cues and detection accuracy between individuals lying about past activities and future intentions, and found a higher accuracy rate in determining false intent, although this may have been attributed to differences in how the observers scored the transcripts. One way to examine this effect explicitly using the current approach would be to present truthful intent participants with information about the mock crime without actual participation in it or possessing information regarding the future actions and compare the responses with those who committed the mock crime.

It might also be possible to add a third condition in which participants complete the mock crime in terms of obtaining the fuse lighter but are not told that it is to be delivered to a contact. Martin et al. (2011) found physiological differences in several parameters, including pupil diameter, between individuals who planned to participate in a malicious event after passing through screening, without first committing a mock crime or attempting to smuggle an illegal device, and innocent participants. Comparisons of the blink behaviors of this group with the group that intends to meet a contact could provide a direct comparison between lying about past actions and false intent. The findings presented here serve as an initial step toward determining the ability of using physiological measures to determine false intent as opposed to lying about previous acts.

### Conflict of interest statement

The author declares that the research was conducted in the absence of any commercial or financial relationships that could be construed as a potential conflict of interest.
